# Effects of interferential current transcutaneous electrical sensory stimulation in patients with severe dementia and dysphagia in long-term care facilities

**DOI:** 10.1186/s12877-025-05912-x

**Published:** 2025-04-24

**Authors:** Yoshiko Hara, Ayako Nakane, Yu Yoshizumi, Kazuharu Nakagawa, Kohei Yamaguchi, Kanako Yoshimi, Haruka Tohara

**Affiliations:** 1 Department of Dysphagia Rehabilitation, Division of Gerontology and Gerodontology, Graduate School of Medical and Dental Sciences, Institute of Science Tokyo, 1-5-45, Yushima, Bunkyo, Tokyo, 113-8549 Japan; 2 Dentistry & Oral Surgery, Japan Community Health-care Organization Tokyo Shinjuku Medical Center, Tokyo, Japan; 3 Oral Surgery, Saitama Red Cross Hospital , Japanese Red Cross Society, Saitama, Japan

**Keywords:** Cough frequency, Cough latency time, Functional oral intake scale, Oral calorie intake, IFC-TESS

## Abstract

**Background:**

The aim of the study was to ascertain the efficacy of interferential current-transcutaneous electrical sensory stimulation (IFC-TESS) in treating patients with severe dementia accompanied by dysphagia who reside in long-term care facilities.

**Methods:**

We conducted a comparative intervention study. Forty-four patients with severe dementia and dysphagia in a long-term care facility were administered IFC-TESS for 15 min twice daily, 5 days a week. The clinical findings before and after 3 weeks of intervention were compared.

**Results:**

The study participants showed significant improvement in oral calorie intake after the intervention (*p<*0.05). The oral calorie intake indicated an improved nutritional state.

**Conclusions:**

IFC-TESS may be effective in improving oral intake by patients with dementia.

**Trial registration:**

The trial was registered at UMIN-CTR under the identifier UMIN000032262 (Registration date: 16/04/2018).

## Background

This study represents a sub-analysis of previously reported findings, focusing on patients with severe dementia. Individuals with dementia often present with swallowing difficulties [1]. Approximately 80% of institutionalized older adults experience dysphagia as dementia progresses, and approximately 50% develop pneumonia [2]. Thus, it can be concluded that the terminal stage of dementia is accompanied by dysphagia and pneumonia [2]. Caregivers at institutions play a significant role in the management of older adults with dementia under these circumstances; however, the presence of dementia makes training (instructional movements) for swallowing disorders difficult. Thus, strategies to overcome the current challenges in dysphagia rehabilitation among older adult patients with dementia in long-term care facilities are essential and warrant discussion.

Cervical interferential current transcutaneous electrical sensory stimulation (IFC-TESS) has, when necessary, been used to treat patients with dementia residing in long-term care facilities with Mini-Mental State Examination (MMSE; includes mild cognitive impairment) [3] scores of ≤ 27. Previous studies have reported a decrease in cough latency time, an improvement in cough frequency and nutritional calorie intake, and a contribution to improved laryngeal sensation [4]. However, the effect of rehabilitation using interference waves on patients with severe dementia who have dysphagia remains unclear. Therefore, in this study, we aimed to clarify the effect of IFC-TESS on patients with severe dementia who have chronic dysphagia (Table 1).

**Table 1:** Comparison with the study [4]

Aspect	Previous Study	Current Study
Patient Group	Patients with dementia and dysphagia	Patients with severe dementia and dysphagia
Primary Outcome	Cough latency	Cough latency
Secondary Outcomes	Cough frequency, FOIS Score,	Cough frequency, FOIS Score,
	oral calorie intake	oral calorie intake
Key Findings	Significant improvement in all outcomes	Significant improvement in oral calorie intake only

In the current study, the same dataset (UMIN000032262) was used; however, the focus was on a specific subgroup

## Methods

Details of the study methodology, including participant recruitment, study design, sample size calculation, outcomes, and statistical analysis, have been described in a previous publication [4].

### Participants

**Fig. 1:**
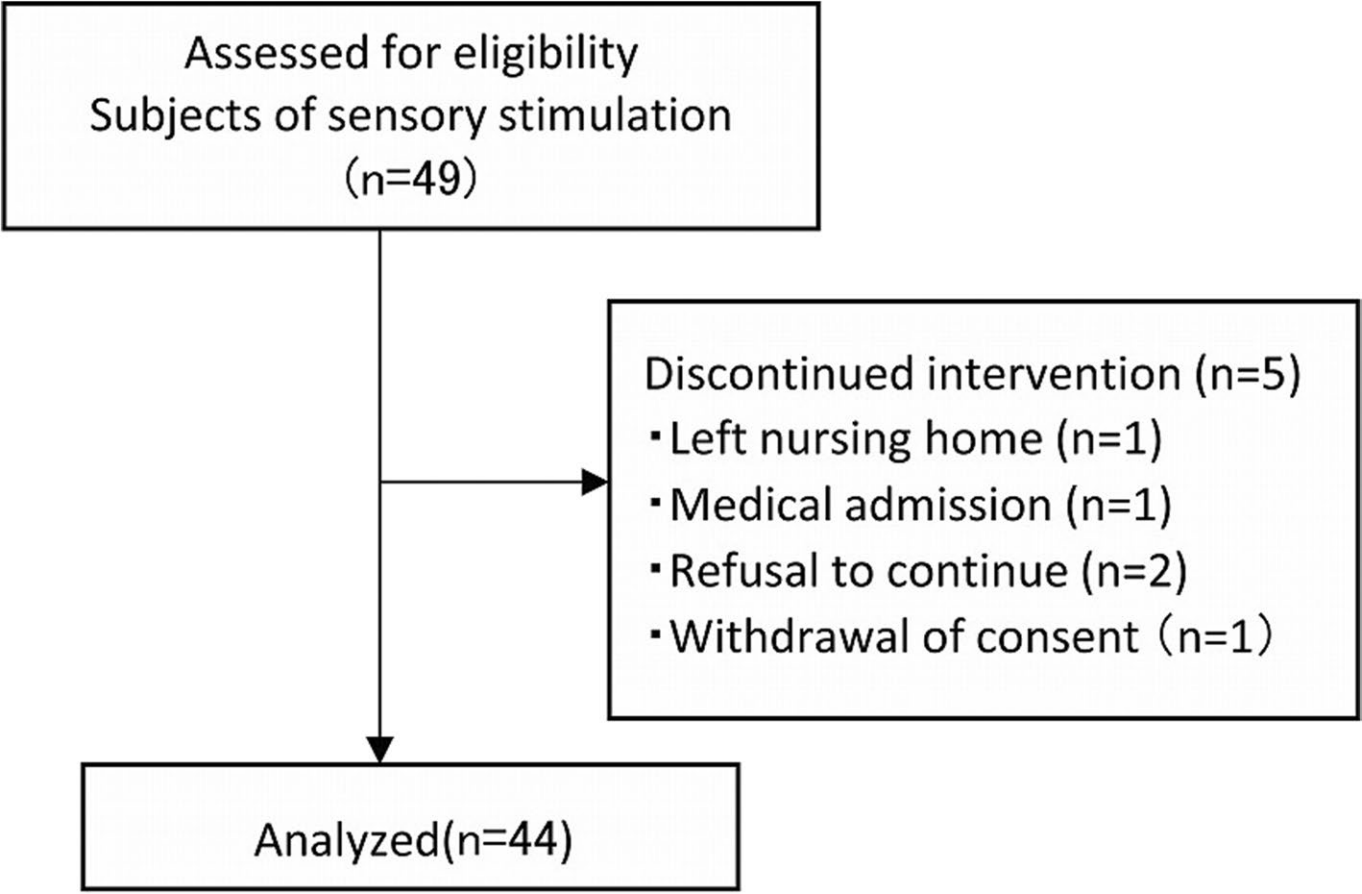
Flowchart of this intervention study. Participants were recruited with consent from facility residents. Some of them were excluded based on exclusion criteria. Interference wave sensory stimulation was initiated for the remaining 49 participants evaluated for eligibility at the three facilities. Forty-four were included in the final analysis after excluding five who were unable to perform for more than three days during the intervention

A pre- and post-intervention comparative study was conducted. The study participants were older adults with dysphagia who were residing in eight long-term care facilities.Among the 49 patients with severe dementia and MMSE scores of ≤ 10 enrolled in this study, 44 (38 women and 6 men; average age, 85.5 ± 11.4 years) were included in the final analysis (Fig. 1). The participants’ history and the study’s exclusion criteria are shown in Fig. 1. The study was conducted between December 2018 and September 2019. This study was conducted according to the tenets of the Declaration of Helsinki and was approved by the Ethics Review Committee at the Faculty of Dentistry, Science Tokyo (approval number: D2018-005). Written and verbal informed consents were obtained from all patients or their families before they were included in this study, and the trial was registered at UMIN-CTR under the identifier UMIN000032262.

### Study design and evaluation

IFC-TESS (Gentle Stim^®^; Food Care Co. Ltd., Kanagawa, Japan) was administered twice daily for 5 days a week for 3 weeks. It was administered continuously for 15 min in the morning and afternoon. The output current during treatment was set at 2.0–3.0 mA to prevent pain based on findings from previous studies [5, 6], and Gentle Stim electrodes were attached to the neck [4].

The cough reflex was evaluated to determine the efficiency of the intervention [7]. In the cough test, patients orally inhaled a mist of citrate saline using an ultrasonic nebulizer for 1 min. Cut-offs of five or more coughs were considered negative (normal), whereas less than four coughs were classified as positive [7]. The concentration of citric acid used was 1.0 w/v% [7] Based on the reference [8], a cough latency time of < 60 s was within the normal range. Therefore, the cough latency time was recorded at the time of the first cough and was measured up to a maximum duration of 1 min.

Information regarding the demographic characteristics, such as the age and sex of the participants and the etiology of dysphagia, was collected from the facility records. The body mass index (BMI) and Mini-Nutritional Assessment (MNA) scores were considered parameters of nutritional status. The MMSE scores were used to assess cognitive function. Moreover, the Barthel index (BI) was adopted as an indicator of basic activities of daily living (ADL) [9].

### Sample size

The sample size was calculated by comparing cough latency time, the primary outcome, before and after the intervention. The effect size in the present study was set to 0.46 based on that of the primary outcome reported in a previous study [6]. The α-value and power were set to 0.05 and 0.8, respectively [10, 11]. Accordingly, the required sample size was 41 participants. However, 44 patients were enrolled, assuming a dropout rate of 10%.

### Outcomes

The cough latency time was the primary outcome [6]. The secondary outcomes were cough frequency, Functional Oral Intake Scale (FOIS) score [12, 13], and oral calorie intake. Each outcome was measured before and 3 weeks after the intervention, and the values were compared.

### Statistical analysis

The Wilcoxon signed-rank test was used to compare the outcomes before and after the intervention. Statistical significance was set at *p*<0.05. Multiple regression analysis was performed using the differences in cough latency time, cough frequency, FOIS score, and oral calorie intake before and after the intervention as dependent variables. Independent variables included age, which is a confounding factor for swallowing function and cough reflex; sex, which showed a significant difference in cough reflex at baseline (Table 2); and MNA score, an indicator of nutritional status.

**Table 2:** Baseline characteristics of the patients stratified by sex

**Characteristics**	**Total (*n***=**44)**	**Women (*n***=**38)**	**Men (*n***=**6)**	**Difference in sex**
Age (years)	85.5 ± 11.4	86.2 ± 11.9	81.3 ± 7.0	0.061
BMI (kg/m^2^)	19.4 ± 3.3	19.1 ± 3.4	20.8 ± 2.1	0.16
MNA score	16.5 ± 3.6	16.4 ± 3.6	18.5 ± 2.6	0.131
BI	11.8 ± 17.9	11.6 ± 18.3	13.3 ± 16.6	0.881
MMSE score	2.1 ± 3.2	2.1 ± 3.4	2.3 ± 1.6	0.231
Cough latency time (s)	14.1 (7.5–57.0)	11.2 (7.2–41.5)	60.0 (44.3–60.0)	0.01*
Cough frequency (n/min)	5.0 (0.5–5.0)	5.0 (3.3–5.0)	0.0 (0.0–5.0)	0.018*
FOIS	5.0 (4.0–5.0)	5.0 (4.0–5.0)	5.0 (4.3–5.0)	0.582
Nutritional oral intake (kcal/day)	1030.0 (800.0–1300.0)	1005.0 (750.0–1300.0)	1310.0 (1073.8–1400.0)	0.113
Comorbidities, n (%)				
Stroke	10 (22.7)	8	2	
Cognitive disorder	29 (65.9)	25	4	
Parkinson disease	1 (2.3)	1	0	
Others, Unknown	4 (9.1)	4	0	

Age, BMI, MNA score, BI, MMSE score are given as mean ± SD or n (%)

Cough latency time, Cough frequency, FOIS, Nutritional oral intake are given as median (IQR) for Wilcoxon signed-rank test

*Abbreviations: BI* Barthel index, *BMI* body mass index, *FOIS* Functional Oral Intake Scale score, *MNA* Mini-Nutritional Assessment, *MMSE* Mini-Mental State Examination, *SD* standard deviation, *IQR* interquartile range

*statistically significant

## Results

Table 2 shows the comparison between the ages, BMI, MNA scores, BI, MMSE scores, cough latency times, cough frequencies, FOIS scores, and oral calorie intake of the participants, highlighting the participant’s sex-related differences at baseline. Table 3 presents a summary of the results of the cough reflex and nutritional intake before and 3 weeks after the IFC-TESS intervention. The oral calorie intake significantly improved after the intervention (*p*=0.002). Furthermore, multiple regression analysis revealed that none of the factors affected the oral calorie intake after adjusting for age, sex, and MNA scores (*p*<0.01, Table 4).

**Table 3:** Comparison of the cough reflex and nutritional intake before and after the intervention

	**Before (*n***=**44)**	**After 3 weeks (*n***=**44)**	*p***-value**	**Effect size**
Cough latency (s)	14.1 (7.5–57.0)	7.9 (3.4–44.3)	0.067	0.548
Cough frequency (n/min)	5.0 (0.5–5.0)	5.0 (0.5–5.0)	0.585	0.334
FOIS score	5.0 (4.0–5.0)	5.0 (4.0–5.0)	> 0.999	0.433
Nutritional oral intake (kcal/day)	1030.0 (800.0–1300.0)	1075.0 (872.3–1300.0)	0.002*	0.776

Data are presented as median (IQR) for the Wilcoxon signed-rank test

*Abbreviations*: *FOIS* functional oral intake scale, *IQR* interquartile range

*statistically significant

**Table 4:** Comparison by difference before and after the 3-week IFC-TESS

Dependent Variable	Independent Variable	B (95%Cl)	β	*p*-value	VIF	Adjusted R^2^
Cough latency	Age(year)	− 0.525to-0.761	0.056	0.714	1.023	
	Sex	1.973 to44.994	0.338	0.033*	1.064	0.051
	MNA	− 1.854to2.293	0.032	0.832	1.044	
Cough frequency	Age(year)	− 0.044to0.073	0.073	0.614	1.023	
	Sex	1.082 to4.974	0.459	0.003*	1.064	0.140
	MNA	− 0.213to0.162	− 0.040	0.783	1.044	
FOIS	Age(year)	− 0.005to-0.006	0.016	0.913	1.023	
	Sex	− 0.404to-0.020	− 0.341	0.031*	1.064	0.056
	MNA	− 0.009to-0.028	0.161	0.294	1.044	
Nutritional oral intake	Age(year)	− 1.217to3.936	0.165	0.293	1.023	
	Sex	− 95.208to77.143	− 0.033	0.833	1.064	− 0.003
	MNA	− 2.799to13.815	0.209	0.188	1.044	

Data are given for the multiple regression model

*Abbreviations: B* unstandardized coefficient, *95% CI* 95% confidence interval, *β* standardized coefficient, *VIF* variance inflation factor, *R2* Coefficient of determination, *BMI* body mass index, *MNA-SF* Mini-Nutritional Assessment Short Form, *BI* bathel index, *MMSE* Mini-Mental State Examination

*statistically significant

## Discussion

The present study represents a sub-analysis of previously reported findings. While the primary outcome of cough latency time did not show a statistically significant improvement in patients with severe dementia, the consistent improvement in oral calorie intake suggests that IFC-TESS may benefit patients across different stages of dementia severity.

### Participant characteristics

Forty-four patients with severe dementia accompanied by dysphagia who had MMSE scores of ≤ 10 were included in this study (Fig. 1). The average age of the study participants was 85.5 ± 11.4 years.

The baseline BI and MMSE scores were 11.8 ± 17.9 and 2.1 ± 3.2, respectively. The participants’ level of independence was low. Hence, they required dietary assistance. Misconception, memory, and visuospatial ability were low for these patients. In general, patients with dementia tend to have a low BMI [14]. The nutritional status of patients with severe dementia accompanied by dysphagia is likely reduced [15]. Oral intake significantly reduces during the end stage of dementia, and malnutrition is considered inevitable. The participants had severe dementia, and their nutritional status was not good with an MNA score of 16.5 ± 3.6.

A comparison of the cough reflexes of men and women before the intervention revealed that women have significantly shorter cough latency (*p*=0.01) and significantly higher cough frequency than men (*p*=0.018). Women generally have a lower cough threshold [16]. In this study, the women were, on average, 4.9 years older than men; nevertheless, they had faster cough reflexes than men before the intervention.

### The effect of IFC-TESS on oral calorie intake

IFC-TESS significantly increased oral calorie intake regardless of dementia severity.

Reportedly, factors such as ADL, antecedent disorders, swallowing function, meal assistance, and MNA scores affect survival duration [17]. Dementia is one of the factors that lead to malnutrition and overcoming malnutrition is important.

Patients with chronic neurological diseases, including dementia, are at constant risk of malnutrition. This can lead to respiratory muscle atrophy and an increased risk of aspiration pneumonia. Furthermore, continuous electrical stimulation, such as IFC-TESS, may help mitigate these risks by improving sensory function and facilitating safer oral intake.

In the present study, the oral calorie intake improved significantly (*p*=0.002) after IFC-TESS, (Table 3). Calorie intake generally increases as food texture improves [18]. However, in this study, the FOIS scores and the oral environment, such as the pharyngolaryngeal sensory function remained unchanged during the intervention period, suggesting that the increased oral calorie intake was due to a higher total intake rather than a change in the diet texture.

Impairment of swallowing function can also occur and is one of the causative factors of malnutrition as patients transition from mid- to end-term dementia. The impairment may be due to bolus formation, poor transport to the pharynx, decreased pharyngeal clearance, and delayed swallowing reflex. In addition, the impairment may be a manifestation of the pathological progression of Alzheimer’s disease during the pharyngeal stage [19].

A series of swallowing movements are pattern-outputted by the swallowing central pattern generator and are believed to be activated by input from the motor and afferent sensory neurons [20]. Methods for improving pharyngeal sensation can be broadly classified as physical and chemical stimulation. IFC-TESS and oral care [21, 22] are classified as physical stimuli acting on peripheral nerves, whereas peripheral stimulation is considered afferent stimulation in the central nervous system [6]. Both IFC-TESS and oral care are effective interventions and are complementary. However, when cognitive function declines, daily self-care, such as oral care, may be hindered, and patients may refuse oral care. Therefore, they may become dependent on assistance from institutional caregivers [23, 24]. In addition, it is difficult to receive specialized care, and not all older adults can receive it [23]. Older adults with severe dementia have poor access to the pharynx, resulting in a decrease in their oral intake, which leads to a decrease in self-cleaning, making it difficult to maintain pharyngeal function [19]. On the other hand, IFC-TESS is easy to operate and can be performed by institutional caregivers under the guidance of an expert. It is a useful method that allows non-experts to provide support for swallowing rehabilitation for patients with severe dementia who cannot perform swallowing training on their own. In the present study, IFC-TESS reduced the threshold of sensory nerves due to afferent input from the superior laryngeal nerve [20], which may lead to improved oral calorie intake. The oral calorie intake in this study increased by 45 kcal over 3 weeks. This increase cannot be ignored for long-term intervention.

### Interpretation of cough latency results

Significant relationships have been found between the rate of pneumonia and laryngeal sensory deficits leading to silent aspiration [25]. The cough reflex does not usually change with age [26], but it may decrease in older adults with chronic diseases [27]. The cough reflex sensitivity is significantly reduced in older adults with adequate respiratory function [28]. All participants in this study had chronic diseases; however, the cough reflex was maintained, and the baseline cough frequency was relatively good. Thus, no statistically significant improvement was observed (*p*=0.067). Other possible reasons for this observation include cellular and network dysfunction and impaired neurotransmission [29] in severe dementia. Therefore, the effects of IFC-TESS may require more than 3 weeks to be revealed.

### Future research direction

IFC-TESS has been shown to help facilitate immediate swallowing and protect airway protection through the brain stem [30]. It also improves the cough reflex and oral intake as short-term effects when combined with ordinary swallowing rehabilitation [6]. Continuous electrical stimulation activates the peripheral sensory nerves, induces changes in plasticity in the cerebral cortex, and changes the excitability of the pharyngeal motor cortex [31]. IFC-TESS increases nutritional intake in patients with severe dementia who often experience a continuous decline in their condition. Notably, the duration of this study was 3 weeks. The cough latency time did not significantly improve during this period; nevertheless, it is possible that cough reflex and oral intake may improve if the period is extended. Hence, in future studies, a long-term intervention period should be considered.

### Limitations

The study has some limitations. It is possible that unmeasured confounding factors existed and that the adjusted R-squared values were relatively low (< 0.5).

Additionally, the durability of the effect after the intervention was not evaluated in the present study. Moreover, the efficacy of IFC-TESS was unknown, as patients with dysphagia who only received tube feeding were excluded from this study. Finally, the types of dementia that could not be classified as unknown cases were included in the present study.

## Conclusions

In a long-term care facility, the oral intake of patients with severe dementia accompanied by dysphagia improved after receiving cervical IFC-TESS. Therefore, IFC-TESS is expected to be an effective approach for patients with dementia regardless of its severity.

## Data Availability

The datasets used and/or analyzed during the current study are available from the corresponding author (Ayako Nakane, Department of Dysphagia Rehabilitation, Division of Gerontology and Gerodontology, Graduate School of Medical and Dental Sciences, Institute of Science Tokyo, Tokyo, Japan, email address: a.nakane.swal@tmd.ac.jp) on reasonable request.

## Abbreviations

ADL: Activities of daily living
BI: Barthel index
BMI: Body mass index
FOIS: Functional Oral Intake Scale
MMSE: Mini-Mental State Examination
MNA: Mini-Nutritional Assessment
SD: Standard deviation
